# A 60-year-old female with left shoulder pain

**DOI:** 10.4103/1817-1737.65039

**Published:** 2010

**Authors:** Hamid Shaaban, John W. Sensakovic

**Affiliations:** St Michael’s Medical Center (Seton Hall University), Infectious Diseases Division, 111 Central Avenue, Newark, NJ 07102

A 60-year-old Caucasian female with past medical history of dyslipidemia presented with left shoulder pain described as constant, dull in nature, radiating down her left arm and not associated with motion. She denies any history of trauma. No fever, chills, redness or swelling or weight loss. She had a smoking history. She denied any alcohol or illicit drug use. She worked as a clinical psychologist and she was happily married. She admitted to having a chronic cough with intermittent arthralgias and morning stiffness. Her physical examination was unremarkable. Her complete blood count and comprehensive metabolic profile was unremarkable and revealed no evidence of any anemia or renal disease. Urinalysis revealed hematuria but no casts or proteinuria.

CT scan of the chest [Figure 1] was done. Pathology of CT-guided biopsy specimens revealed chronic granulomatous inflammation without any neoplastic process. ESR was 73 and C-ANCA was positive. Blood tests such as rheumatoid factor levels, anti-nuclear antibody titers, P-ANCA, anti-myeloperoxidase and protease-3 abs were negative for other collagen vascular diseases.

**Figure 1:**
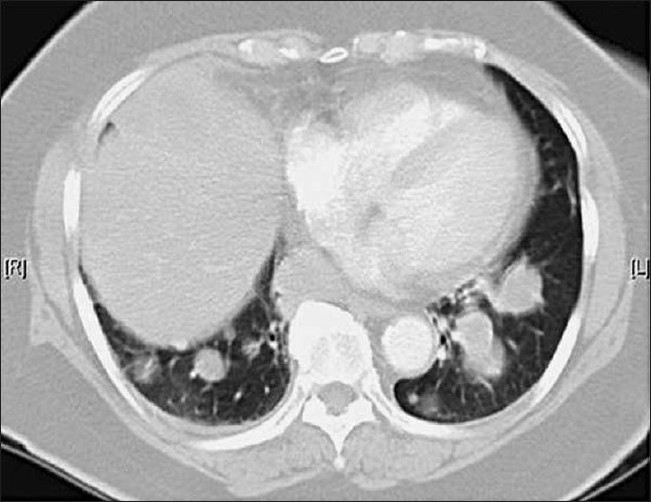
Computed tomography scan of chest

## Questions

1. What are the findings on the computed tomography (CT) scan of the chest?
2. What other serologic tests would you order?

## Answers

1. CT scan confirmed the presence of a 9.5 × 8.3-cm right middle lobe mass, 3.7 × 2.9-cm and 3.1 × 2.2-cm right lower lobe masses, a 3.6 × 2.0-cm left upper lobe mass and many diffuse smaller nodules throughout the lung fields.
2. ESR was 73 and C-ANCA was positive. Blood tests such as rheumatoid factor levels, anti-nuclear antibody titers, P-ANCA, anti-myeloperoxidase and protease-3 abs were negative for other collagen vascular diseases.

## Course of the Patient

Diagnosis of Wegener’s granulomatosis was made and the patient was started on corticosteroids and oral cyclophosphamide 1 mg/kg/day, which resulted in resolution of pulmonary nodules. Subsequently patient had resolution of her previously persistent left shoulder pain.

## Discussion

Shoulder pain is the most common presenting symptom of Pancoast syndrome, related to the involvement of the lower trunk of the brachial plexus, the first three ribs, the vertebrae, or the parietal pleura. Horner’s syndrome, recurrent laryngeal and phrenic nerve involvement are less common features. Pulmonary manifestations, such as cough, hemoptysis and dyspnea, occur less often than with centrally located endobronchial lesions.[1]

*Wegener’s granulomatosis* presenting as Pancoast syndrome has only once been previously reported in the literature. ‘Classic’ Wegener’s granulomatosis is a form of systemic vasculitis that primarily involves the upper and lower respiratory tracts and the kidneys. A ‘limited’ form, with clinical findings isolated to the upper respiratory tract or the lungs, occurs in approximately one-fourth of cases.

Up to one-third of patients with Wegener’s granulomatosis who present with pulmonary involvement may not have respiratory symptoms. Other organ systems that may become involved in addition to the kidney include:[2] joints (myalgias, arthralgias, arthritis), eyes (conjunctivitis, episcleritis, uveitis), skin (vesicular, purpuric and hemorrhagic lesions), nervous system (mononeuritis multiplex, cranial nerve abnormalities, external ophthalmoplegia, tinnitus, hearing loss), heart (pericarditis, myocarditis, conduction system abnormalities) and less commonly, the gastrointestinal tract, subglottis or trachea, lower genitourinary tract (including the prostate or ureter), parotid glands, thyroid, liver or breast

Nonspecific complaints of fever, anorexia, weight loss and malaise may accompany upper or lower airway disease.

Laboratory findings are generally nonspecific in Wegener’s granulomatosis including those with pulmonary involvement. Common abnormalities include leukocytosis, thrombocytosis (>400,000/mm^3^), elevated ESR and anemia of chronic disease. The urinalysis is normal in limited Wegener’s granulomatosis. However, patients with renal involvement typically show an elevation in the BUN and plasma creatinine concentration, mild to moderate proteinuria and RBC, WBC and cellular/hyaline casts.

Most patients (90%) with Wegener’s granulomatosis have C-ANCA, characterized by autoantibodies directed against serine proteinase 3. In some circumstances, a positive ANCA may provide the only clue to the correct diagnosis and may prompt decisive diagnostic and management decisions.

In addition to its diagnostic utility, the level of the C-ANCA has been used to follow the disease course. Diagnosis of pulmonary masses relies on cytologic, histologic and microbiologic examination of tissue. This is vital because not only does it help in diagnosis but it also impacts on treatment. For apical tumors, percutaneous biopsy is reported as the modality of choice.[3]

Although the initiation of treatment for apical lung lesions was once advocated without the acquisition of a precise histologic diagnosis,[4] the recognition that Pancoast syndrome can be caused by a wide variety of conditions mandates that an accurate diagnosis is made in any pulmonary mass that appears clinically and radiologically to be a bronchogenic carcinoma. CT-guided percutaneous biopsy remains the best modality of choice, but where the diagnosis remains elusive and uncertain, open biopsy is required.
